# Design and Optimization of an Ultrathin and Broadband Polarization-Insensitive Fractal FSS Using the Improved Bacteria Foraging Optimization Algorithm and Curve Fitting

**DOI:** 10.3390/nano13010191

**Published:** 2023-01-01

**Authors:** Yaxi Pan, Jian Dong

**Affiliations:** School of Computer Science and Engineering, Central South University, Changsha 410083, China

**Keywords:** improved bacteria foraging optimization, frequency selective surface, fractal structure, curve fitting

## Abstract

A frequency-selective surface (FSS) optimization method combining a curve-fitting technique and an improved bacterial foraging optimization (IBFO) algorithm is proposed. In the method, novel Koch curve-like FSS and Minkowski fractal islands FSS were designed with a desired resonance center frequency and bandwidth. The bacteria foraging optimization (BFO) algorithm is improved to enhance the performance of the FSS. A curve-fitting technique is provided to allow an intuitive and numerical analysis of the correspondence between the FSS structural parameters and the frequency response. The curve-fitting results are used to evaluate the fitness function of the IBFO algorithm, replacing multiple repeated calls to the electromagnetic simulation software with the curve-fitting equation and thus speeding up the design process. IBFO is compared with the classical BFO algorithm, the hybrid BFO-particle swarm optimization algorithm (BSO), and the artificial bee colony algorithm (ABC) to demonstrate its superior performance. The designed fractal FSS is fabricated and tested to verify the experimental results. The simulation and measurement results show that the proposed FSS has a fractional bandwidth of 91.7% in the frequency range of 3.41–9.19 GHz (S, C, and X-bands). In addition, the structure is very thin, with only 0.025λ and 0.067λ at the lowest and highest frequencies, respectively. The proposed fractal FSS has shown stable performance for both TE and TM polarizations at oblique incidence angles up to 45°. according to simulations and measurements.

## 1. Introduction

Frequency selective surface (FSS) is typically a resonant unit printed on a dielectric substrate and placed in a periodic pattern. Near the single-cell resonance, FSS shows entire reflection or transmission qualities. It has been employed in various applications, including radomes, sub reflectors for dual-band reflector systems [1,2,3,4,5], and FSSs with high-efficiency transmission and low-scattering properties [6,7,8]. Many FSSs are proposed using various topologies and methodologies. A standard FSS is made up of a regularly structured surface and resonant elements, such as slot and patch elements. Bandpass FSSs are designed with slot components, whereas bandstop FSSs are implemented with patch elements [9,10]. New requirements for the potential implementation of multi-band and broadband FSSs, which can be achieved through a variety of innovative technologies and advanced materials, have been proposed [11,12,13,14,15]. The fractal structure offers a novel research approach for the creation of broadband and multi-band antennas [16,17]. Fractal structures are a class of complex geometric structures that are self-similar, and their geometry and dimensionality have a significant impact on their operational resonant frequencies. Fractal properties, such as space-filling, self-similarity, and unlimited complexity, allow them to be miniaturized, broadband, and multi-band [18,19]. FSSs with a fractal structure has piqued the interest of researchers for the advantages in the terms listed above [20]. A technique is proposed to mix fractal FSS with single- and double-layer coatings that can be applied to thin broadband absorber layers [21]. Researchers have suggested a fractal-based FSS and compared the bandwidth at various fractal levels [22]. The results indicate that employing fractal elements results in a larger bandwidth than without them. A Minkowski fractal island-based FSS is proposed, and the FSS is shown to have exceptional angular stability and polarization insensitivity [23].

However, in the process of FSS design and optimization, parameter scanning with electromagnetic (EM) simulation software is frequently time-consuming. Thus researchers have begun to experiment with optimization algorithms to optimize the FSS structural parameters [24,25,26]. The optimization algorithm-based approach requires repeated calls to the EM simulation software over the course of the optimization process, which could take hours or even days as the number of populations and iterations increases. Several researchers have proposed optimization methods using machine learning for a surrogate model [27]. However, there are some limitations to this approach, such as the difficulty of determining the neural network model parameters, the requirement for a large training set, and the time-consuming nature of training the model. This gap has been the impetus for the current work. The curve-fitting method is able to address the above challenges to some extent [28,29]. The method provides graphical analysis, graphically and digitally, of the correspondence between structural parameters and frequency response [30]. The most significant advantage of curve fitting is forming equations that best fit the experimental data and then predicting the design performance based on these equations.

In this paper, a novel Koch curve-like and Minkowski fractal island structure are used to design an ultrathin and broadband polarization-insensitive fractal FSS. Despite very small changes in resonant frequency and bandwidth results for different values of the oblique incident angle, the proposed FSS geometry showed high angular stability. An improved bacteria foraging optimization (IBFO) is proposed for the optimization and design of fractal FSS. BFO is improved by optimizing the step size of the bacteria foraging optimization (BFO) algorithm, updating the bacterial positions using the concept of updating positions with the particle swarm optimization (PSO) algorithm, selecting unique generation individuals for crossover operations using the roulette wheel method, and using adaptive migration probabilities. The curve-fitting technique is used to determine the correspondence between the FSS structural parameters and the frequency response, with the results subsequently used to evaluate the fitness function of the IBFO algorithm. Then, the IBFO can accurately determine the parameters of the target frequency response without the need to run the electromagnetic simulation, which greatly improves the efficiency of the design.

The rest of the paper is organized as follows. In Section 2, FSSs based on Minkowski fractal islands and Koch curve are proposed. In addition, the BFO algorithm is improved to enhance the optimization performance. The curve-fitting technique is used to establish the correspondence between the structural parameters of the FSS and the frequency response. In Section 3, the fitness equation is established to optimize the fractal FSS, and the optimization results are compared with other algorithms. Section 4 gives experimental validation of the optimized FSS. The designed fractal FSS is fabricated and measured. Section 5 concludes the paper.

## 2. Design of the Proposed FSS Using IBFO and Curve Fitting

### 2.1. Geometrical Structure

**Figure 1:**
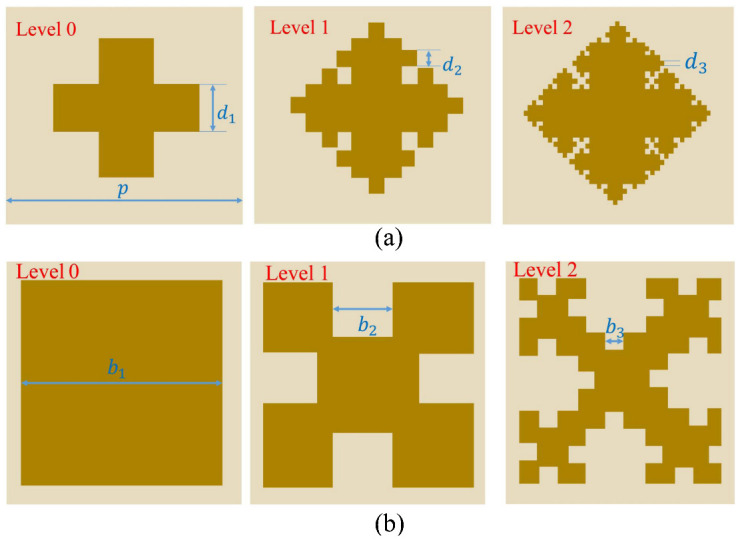
Koch curve-like and Minkowski geometry fractal iterations for levels 0, 1, and 2. (**a**) Koch curve-like fractal structure, (**b**) Minkowski fractal island structure.

A fractal antenna is a structure generated by the repetitive assembly of self-similar geometric shapes in particular proportions. The fractal formations include Sierpinski geometry, Koch curve, Minkowski geometry, curved flow geometry, Giuseppe Peano, and Hilbert curve [31,32]. Having a self-similarity and space-filling nature, it resonates at a broad spectrum of frequencies. FSS with fractal structures has a compact design, making them suitable for the design of miniature FSS. In this paper, a broadband FSS using Koch curve-like fractal elements, and Minkowski geometry is designed. Figure 1 shows the iterative generation process of fractal geometry.

A recursive process is used to generate the fractal structure, which includes two fractal parameters: the number of iterations (or levels) and the iteration factor. The proposed FSS’s top layer is fractalized by novel Koch-like curves, which are generated using crossover structures, and then formed by bending the middle part of each original straight line segment into a square in two iterations. The iteration factor $It_{t1}=d_{2}/d_{1}$ for level 1. Similarly, with the iteration factor $It_{t2}=d_{3}/d_{1}$, the final FSS upper metal patch structure is obtained. The fractal is produced by reiterating this procedure an endless multitude of times, whereas a pre-fractal is produced if the recursive process ends after a bounded number of times. To be more accurate, manufacturable fractal items must occur from a truncated generation stage and are thus defined as pre-fractals. The bottom layer is generated recursively using Minkowski island. A straight line (initiator) is used to create Minkowski curve geometry. In each recursion, and then in the previous iteration, a 4-edge generator is applied to the pre-fractal, as shown in Figure 1b. The iteration factors for levels 1 and 2 are $It_{b1}=b_{2}/b_{1}$ and $It_{b2}=b_{3}/b_{1}$, respectively. $d_{1}$, $d_{2}$, $d_{3}$, $b_{1}$, $b_{2}$, and $b_{3}$ are the parameters responsible for the multi-band feature of the FSS.

**Figure 2:**
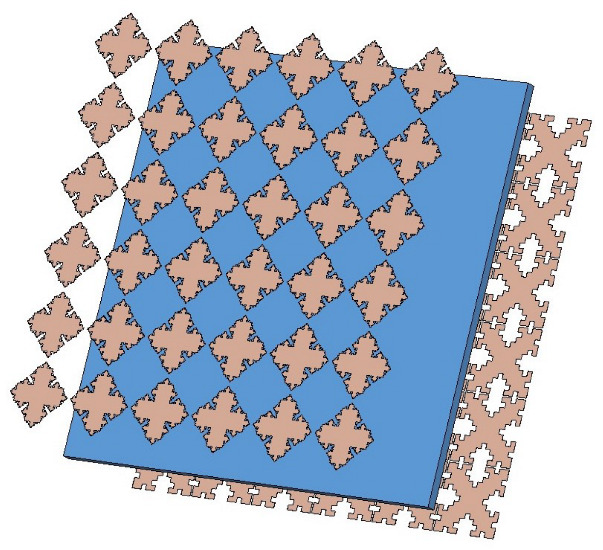
Geometry of the proposed fractal FSS.

The 3D schematic of the proposed fractal FSS is shown in Figure 2. The structure of the proposed FSS consists of a periodic array of patch elements mounted on a fiberglass substrate (FR-4), with a dielectric constant $\varepsilon_{r}=4.3$, loss tangent tanδ=0.025, and the dielectric thickness *h* (as shown in the rectangle in the figure). Koch curve-like fractal elements and Minkowski geometric fractal elements are attached to the upper and lower layers of the dielectric substrate, respectively, and the material is copper (as shown in dark yellow in Figure 1).

**Figure 3:**
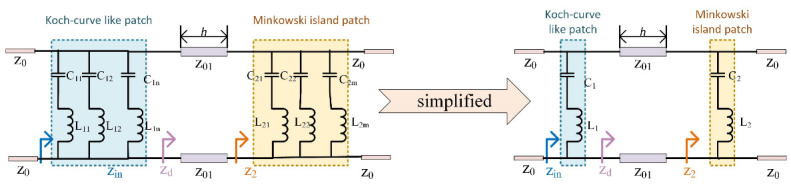
Equivalent circuit of the proposed fractal FSS.

As can be seen in Figure 3, the equivalent circuit of the Koch curve-like patch and the Minkowski island patch structure can be approximated as the series-circuit resonant circuit. The proposed structure can realize a band-stop FSS with dual resonant frequencies. The resonant frequency can be calculated as $f_{c,i}=\frac{1}{2\pi \sqrt{L_{i}C_{i}}}$. The equivalent inductance is dependent upon the length of the patch structure, while the equivalent capacitance is dependent upon the spacing between the patches [9]. Typically, the characteristic impedance of free space is $Z_{0}=377\;ohm$. The characteristic impedance of the dielectric substrate is given by $Z_{01}=\frac{Z_{0}}{\sqrt{\varepsilon_{r}}}$ and the relative dielectric constant is given by $\varepsilon_{r}$. The transmission line model’s input impedance is denoted by $Z_{in}$. The impedances corresponding to the Koch curve-like patch and the Minkowski island patch are [22]:

(1) $$Z_{1}=j\omega L_{1}+\frac{1}{j\omega C_{1}},Z_{2}=j\omega L_{2}+\frac{1}{j\omega C_{2}}$$

(2) $$Z_{in}=Z_{1}\|Z_{d}$$

(3) $$Z_{d}=Z_{01}\frac{Z_{2}+jZ_{01}\tan\left(\beta h\right)}{Z_{01}+jZ_{2}\tan\left(\beta h\right)}$$

where β is the phase constant of the transmission line of the dielectric sample. For oblique incidence, with incidence angle θ and refraction angle ξ, the transmission line parameters must be revised. For TE polarization, the characteristic impedance of the free space and dielectric are $Z_{0}^{*}=Z_{0}\sec\left(\theta \right)$ and $Z_{d}^{*}=Z_{d}\sec\left(\xi \right)$, respectively. Similarly, $Z_{1}^{*}=Z_{1}\sec\left(\xi \right)$. The electric permittivity of free space and the substrate is $\varepsilon_{0}\cos\left(\theta \right)$ and $\varepsilon_{r}\cos\left(\xi \right)$, respectively. For TM polarization, the characteristic impedance of the free space and dielectric are $Z_{0}^{*}=Z_{0}\cos\left(\theta \right)$ and $Z_{d}^{*}=Z_{d}\cos\left(\xi \right)$, respectively. Similarly, $Z_{1}^{*}=Z_{1}\cos\left(\xi \right)$. The electric permittivity of free space and the substrate is $\varepsilon_{0}\sec\left(\theta \right)$ and $\varepsilon_{r}\sec\left(\xi \right)$, respectively. In this case, the input impedance is $Z_{in}^{*}$, $Z_{in}^{*}=Z_{1}^{*}\|Z_{d}^{*}$. Finally, the transmission coefficient of the proposed FSS is represented as:

(4) $$\left|T\right|=1-\left|\Gamma \right|=1-\left|\frac{Z_{in}^{*}+Z_{0}^{*}}{Z_{in}^{*}-Z_{0}^{*}}\right|$$

where |Γ| is the reflection coefficient.

### 2.2. Improved Bacterial Foraging Optimization Algorithm

**Figure 4:**
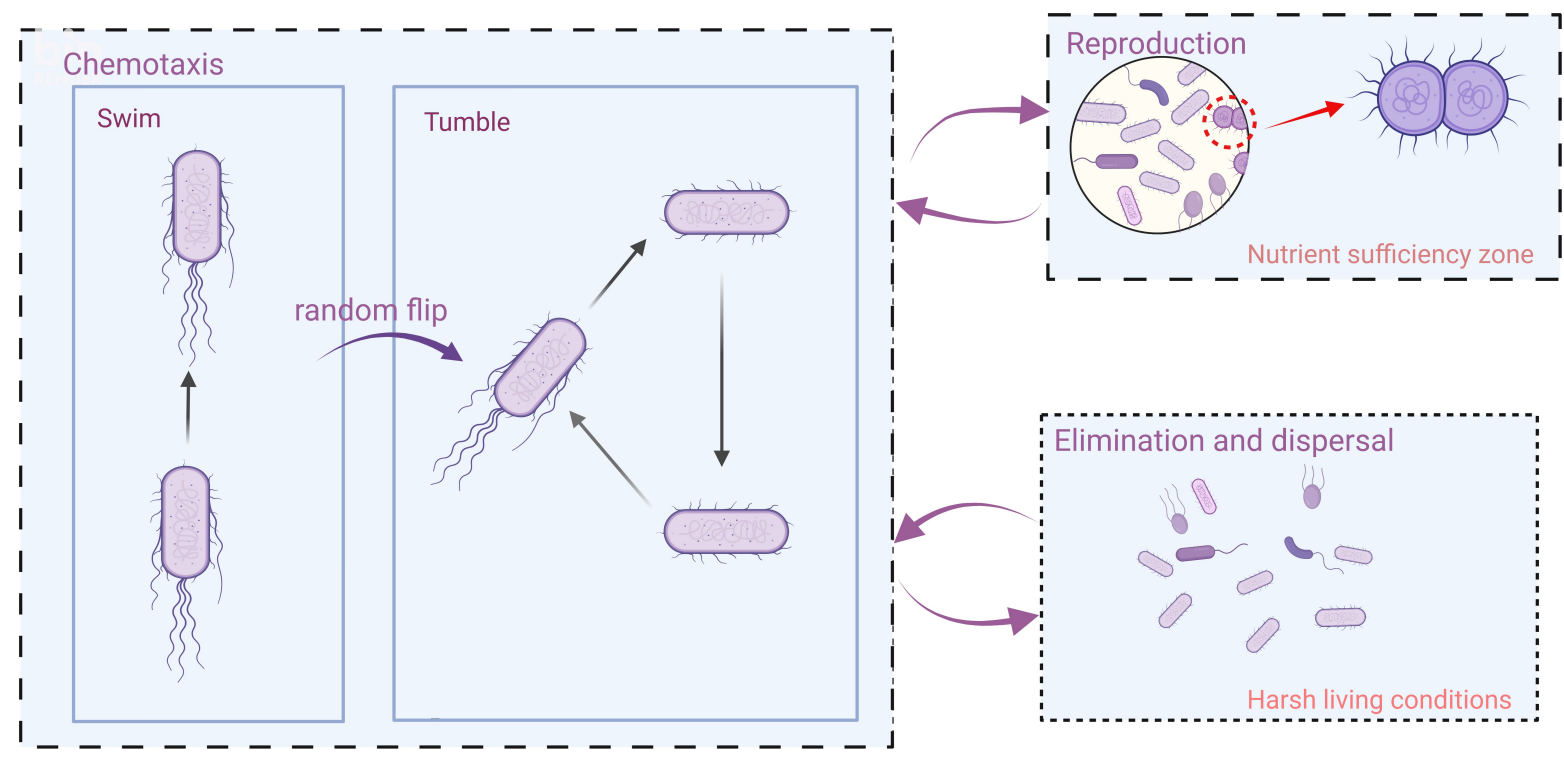
Swimming, tumbling, chemotaxis, reproduction, and elimination and dispersal behavior of *E. coli*.

A novel bionic-like optimization algorithm, the BFO algorithm, has been put out to mimic how Escherichia coli forages for food in the human intestine [33]. The general approach of the BFO to addressing an optimization problem entails creating an original population of candidate solutions, figuring out the fitness function’s value, and then using community interaction to optimize. In the BFO model, the fitness value of the evaluation function, which represents the state of the bacterium in the search space, corresponds to the solution of the optimization problem. Three steps make up the BFO algorithm: chemotaxis, reproduction, and elimination and dispersal [34]. The chemotaxis, reproduction, and elimination and dispersal behavior of *E. coli* is shown in Figure 4.

The algorithm, however, has some drawbacks: (1) The global optimization-seeking capability of the BFO algorithm is strong, but the convergence time is slow. (2) The BFO algorithm’s stability is poor. (3) The BFO algorithm’s replication process produces a subpopulation that is identical to the parent population [35]. The BFO algorithm was improved to address the deficiencies mentioned above.

An adaptive step-size approach was developed for the chemotaxis process. At an early stage of the algorithm, large step sizes are used to accomplish quick global optimization searches. As the algorithm proceeds, step sizes are reduced to enhance the precision of the optimization. The following describes the modified adaptive step size.

(5) $$C_{d}=\frac{C_{max}}{j\cdot k\cdot l}\cdot F\cdot rand\left(\right)$$

where $C_{d}$ denotes the step size of the bacteria in each dimension during the chemotaxis operation. $C_{max}=\left(u_{limit}-l_{limit}\right)$, $C_{max}$ is the initial step of the d−th dimension, and $u_{limit}$ and $l_{limit}$ are the upper and lower limits of the values taken in the dth dimension, respectively. *F* denotes the scaling factor, which takes the value of a random number between [0,0.5]. Where *j*, *k*, and *l* represent the number of current chemotaxes, reproduction, and elimination and dispersal operations, respectively. rand() is the random number of the interval on [0,1]. A random function can make the step size relatively small if the bacteria are near an optimal location early in the process, preventing the bacteria’s search process from skipping the ideal location.

The concept of PSO is borrowed in order to accelerate the speed and capabilities of BFO’s optimization search [36,37]. By comparing the historical optimum $P_{best}$ with the global optimum $G_{best}$, the vector ϕ(i,j+1) responsible for tumbling in chemotactic is improved as follows:

(6) $$\phi \left(i,j+1\right)=w\cdot \phi \left(i\right)+c_{1}\cdot rand\left(\right)\cdot \left(G_{best}-P_{current}\right)+c_{2}\cdot rand\left(\right)\cdot \left(P_{best}-P_{current}\right)$$

where the weight is w=0.9, and $c_{1}$ and $c_{2}$ are the learning rates, $c_{1},c_{2}\in \left[0,2\right]$. $P_{current}$ represents the current location of the i−th bacteria at j+1 chemotaxis step.

The roulette wheel method is used to select individual parents to expand the population’s diversity. The precise procedure is to first determine the probability $p(x_{i})$ of individual *x*, $x\in x_{1},x_{2}\cdots x_{s}$ being chosen (*s* is the population size), $p\left(x_{i}\right)=fit\left(x_{i}\right)/\sum_{j=1}^{s}fit\left(x_{j}\right)$. Then, determine the cumulative probability of each individual, $pc\left(x_{i}\right)=\sum_{j=1}^{i}p\left(x_{j}\right)\left(i=1,2\cdots s\right)$. Finally, produce a random number rand with uniform distribution in the range [0,1], if $rand\leq pc(x_{i})$, $x_{i}$ is selected. If $pc\left(x_{j-1}\right)\leq rand\leq pc\left(x_{j}\right)$, $x_{j}$ is selected. Repeat the above steps S/2 times to obtain the parent individuals for the crossover operation. The crossover formula is shown in Equation (7).

(7) $$x\left(i\right)=\rho x_{best}+\left(1-\rho \right)x$$

where x(i) denotes the new position of bacteria after hybridization; ρ is the random number of the interval on [0,1]; $x_{best}$ is the position of the optimal parent individual; *x* is the initial position of the offspring bacteria *i*.

The adaptive migration probability $p_{ed}^{*}$ is used in this paper to direct bacteria throughout their elimination and dispersal operation. This increases the capacity of bacteria to engage in a global search for the optimal solutions, lowers the likelihood that they will enter local optimum solutions, and ensures that the algorithm will converge quickly while increasing population diversity. The enhanced elimination and dispersal probabilities are displayed below:

(8) $$p_{ed}^{*}=\frac{J_{health}^{i}-J_{health}^{first}}{J_{health}^{last}-J_{health}^{first}}\cdot p_{ed}$$

where $J_{health}^{i}=\sum_{j=1}^{N_{C}}J\left(i,j,k,l\right)$ is a healthiness function measuring the strength of the foraging ability of bacteria *i*, which is expressed as the sum of the fitness values of all locations of bacteria *i* after $N_{c}$ chemotaxis operations. $J_{health}^{first}$ and $J_{health}^{last}$ denote the healthiness of the individual with the largest and smallest $J_{health}$ in the population, respectively. $p_{ed}$ is the original elimination and dispersal probability.

### 2.3. Curve Fitting

Through years of research, researchers have established equations for the correspondence between structural parameters and frequency response for crossover, square slot, square patch, and other defined shapes of FSS. The current study demonstrates that beyond a certain level of complexity, establishing exact mathematical expressions for these geometric shape models among the complex shapes of topologies is a significant challenge. To some extent, the curve-fitting method addresses the aforementioned challenges. This method provides a graphical analysis method for numerically analyzing the behavior of the FSS bandwidth, center frequency, frequency response, and other design parameters. Curve-fitting techniques such as least squares, residuals, and minimum mean square error methods are used to visualize the goodness of fit. Polynomial, exponential, Fourier, Gaussian, and other mathematical operations can be used to appropriately fit the experimental data. The curve-fitting method is distinguished by the fact that the FSS performance is predicted based on the fitted equations by forming the equations that best match the experimental data.

The curve-fitting technique is used in this paper to determine the relationship between the proposed FSS structural parameters and the frequency response. A combined simulation of Matlab 2020 and CST Studio Suite 2020 software is used to determine the dataset of the corresponding frequency response of the FSS by varying different structural parameters. The main structural parameters are the dielectric substrate’s width *p* and height *h*, as well as the structural parameters of the Koch curve-like and Minkowski geometric pre-fractals. We define $S_{21}=-10$ when the transmission coefficient is less than −10 dB within the target bandwidth to characterize the correspondence between the above structural parameters and the frequency response. When the transmission coefficient is greater than −10 dB, the logical value remains constant. The mathematical expression is written as Equation (9):

(9) $$S_{21}=\left\{\begin{array}{c} -10,\;ifS_{21}\leq -10\\ S_{21},\;ifS_{21}>-10 \end{array}\right.$$

At a specific frequency, $S_{21}$ may be much less than −30 or −40 dB, which makes it very difficult to find the extreme value of *S* in Equation (10). Therefore Equation (9) solves this problem very well. Then the function that satisfied the specified frequency band is defined below:

(10) $$S=\frac{-s_{21}\left(f_{1}\right)-s_{21}\left(f_{2}\right)\ldots -s_{21}\left(f_{N}\right)}{N}$$

where *N* represents the samples in the desired bandwidth. The maximum value of *S* is equal to 10. All transmission coefficients within the target bandwidth should be less than −10 dB, i.e., S=10, indicating that the proposed FSS is a frequency response.

The main steps of curve fitting are as follows:

Step 1 Load data (Structural parameters and their corresponding *S*);
Step 2 Create a fit using the fit function, specifying the variables and a model type (Fourier, Polynomial, Gaussian, Exponential, etc.);
Step 3 Calculate the goodness of fit: (1) The sum of squares due to error (SSE) of the corresponding points of the predicted data $predicted_{i}$ and the original data $observed_{i}$; (2) Root mean squared error (RMSE) at the corresponding points of the predicted and original data; (3) Coefficient of certainty (R−square).
  (11) $$SSE=\sum_{i=1}^{n}{\left(observed_{i}-predicted_{i}\right)}^{2}$$
  (12) $$RMSE=\sqrt{\frac{1}{n}\sum_{i=1}^{n}{\left(observed_{i}-predicted_{i}\right)}^{2}}$$
  (13) $$R-square=1-\frac{SSE}{SST}$$
  where SST is the total sum of squares of the difference between the original data and its mean $\bar{observed}$, $SST=\sum_{i=1}^{n}{\left(observed_{i}-\bar{observed}\right)}^{2}$.
Step 4 Observe the evaluation metrics to determine if the fit meets the requirements, and if not, continue to change them. The fitting requirement is satisfied if SSE and RMSE are approximately 0, R−square is approximately 1.

The mathematical expression of the relationship between the FSS structural parameters and the frequency response parameters $S_{i}$ takes the following form

(14) $$\begin{array}{cc} S_{1}\left(d_{1}\right)= & -12.53d_{1}^{4}+170.7d_{1}^{3}-870.6d_{1}^{2}+1969d_{1}-1656\\ S_{2}\left({It}_{t1}\right)= & 5.398-2.803\cos\left({It}_{t1}\cdot 10.33\right)+5.591\sin\left({It}_{t1}\cdot 10.33\right)\\ \; & +0.9933\cos\left(2{It}_{t1}\cdot 10.33\right)+1.326\sin\left({2It}_{t1}\cdot 10.33\right)\\ S_{3}\left({It}_{t2}\right)= & 9.481+0.5574\cos\left({It}_{t2}\cdot 17.86\right)+0.4493\sin\left({It}_{t2}\cdot 17.86\right)\\ \; & -0.04275\cos\left(2\cdot {It}_{t2}\cdot 17.86\right)-0.1943\sin\left({2\cdot It}_{t2}\cdot 17.86\right)\\ S_{4}\left(b_{1}\right)= & -0.1069b_{1}^{3}+4.567b_{1}^{2}-64.9b_{1}-316.7\\ S_{5}\left({It}_{b1}\right)= & 0.853e^{{-\left(\frac{{It}_{b1}-0.2368}{0.03136}\right)}^{2}}+10.02e^{{-\left(\frac{{It}_{b1}-0.1661}{0.2071}\right)}^{2}}+5.309e^{{-(\frac{{It}_{b1}-0.4124}{0.08461})}^{2}}\\ S_{6}\left({It}_{b2}\right)= & 287.4-331.6\cos\left({It}_{b2}\cdot 12.48\right)-272.6\sin\left({It}_{b2}\cdot 12.48\right)\\ \; & +38.6\cos\left(2\cdot {It}_{b2}\cdot 12.48\right)+184\sin\left({2\cdot It}_{b2}\cdot 12.48\right)\\ \; & +15.7\cos\left(3\cdot {It}_{b2}\cdot 12.48\right)-32.57\sin\left({2\cdot It}_{b2}\cdot 12.48\right)\\ S_{7}\left(p\right)= & -0.05\cdot p^{3}-0.03871\cdot p^{2}+0.4896\cdot p+9.347\\ S_{8}\left(h\right)= & 9.57-0.4849\cdot \cos\left(h\cdot 1.475\right)+0.3116\cdot \sin\left(h\cdot 1.475\right)\\ \; & -0.06127\cdot \cos\left(2\cdot h\cdot 1.475\right)+0.1334\cdot \sin\left(2\cdot h\cdot 1.475\right) \end{array}$$

## 3. Performance Assessment of the Proposed Method

**Figure 5:**
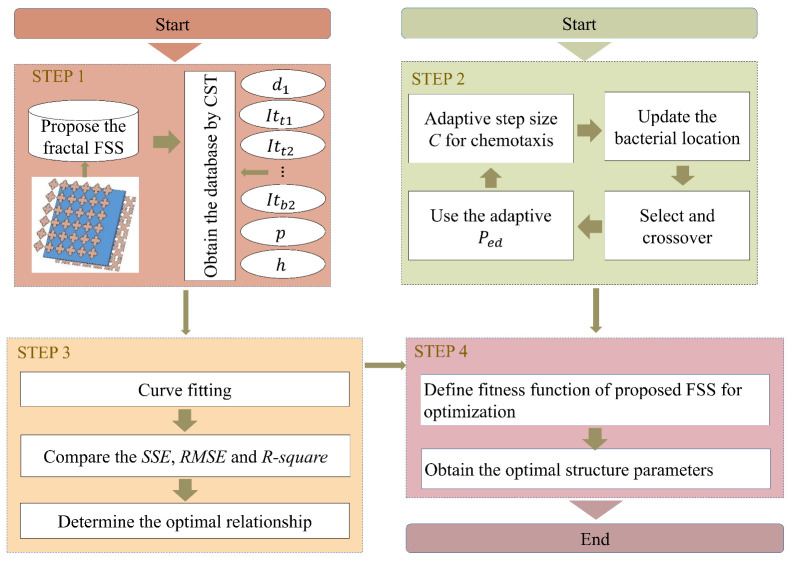
Flowchart for the proposed method.

A fractal FSS is designed and optimized utilizing a combination of curve fitting and the improved bacteria foraging optimization (IBFO) algorithm in Figure 5 to improve the FSS’s performance. As seen in Figure 5, the solution to the issue is to define an appropriate fitness function once the curve fitting is finished. In order to get the parameter values for the proposed FSS with a wider bandwidth, the fitness function is built by taking into account all the essential constraints. According to the analysis in Section 2.3, the fitness function is defined as follows:

(15) $$fitness=min{\left(10-\sum_{i=1}^{n}S_{i}/n\right)}^{2}$$

The effectiveness of IBFO is contrasted with that of other published algorithms, such as the original BFO, hybrid bacteria foraging optimization-particle swarm optimization (BSO) [37], and artificial bee colony (ABC) [38], demonstrating its superiority. In order to achieve optimal results, it is imperative to select the parameters appropriately for the optimization method used. The initial parameters for the IBFO, BFO, BSO, and ABC algorithms are provided in Table 1. The values of the pheromone volatility factor, evaporation rate, and heuristic information in the ABC algorithm are 0.8, 0.2, and 2, respectively.

To make the proposed FSS have a broader bandwidth, the fitness function is created by taking into account all the necessary bounds to reach the optimal parameter values. The smaller the fitness, the better the frequency response of optimized FSS consistent with the desired frequency response. When fitness is approximately 0, the structural parameters’ optimal solution is attained.

It is assumed that the design goal in this paper is to achieve a broadband FSS with a bandwidth of 3.4–9.2 GHz and resonant frequencies of 5.2 and 8.2 GHz, respectively. The boundary conditions of structural parameters to be optimized are shown in Table 2.

**Figure 6:**
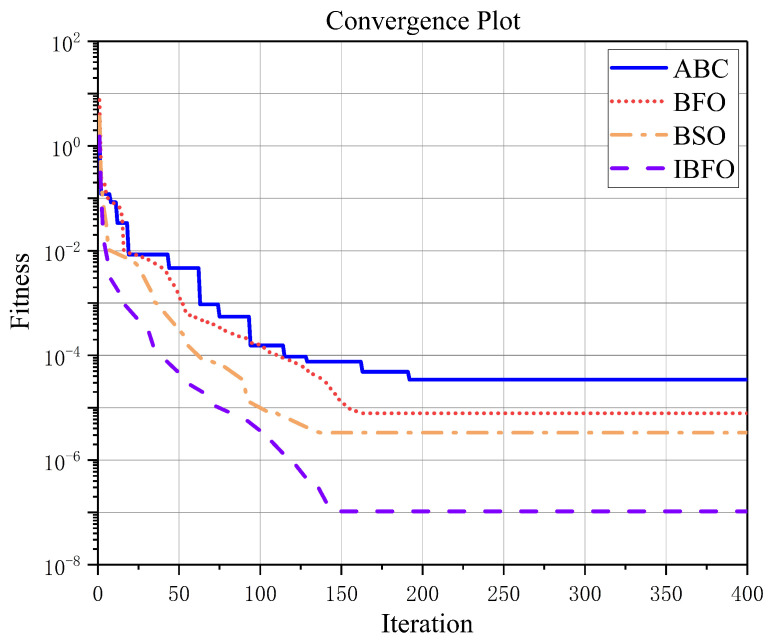
Evolutionary process of average fitness value for different algorithms.

Thirty independent trial runs were performed for all algorithms, with a maximum number of 400 $\left(N_{C}*N_{re}*N_{ed}\right)$ iterations per algorithm. The fitness function is displayed in Equation (15). As compared with the other three algorithms, IBFO is two to three orders of magnitude better in terms of the quality of the solutions based on the average solution, the worst solution, and the standard deviation (the comparative performance is shown in Table 3). In light of the metric for evaluating the best solution, the IBFO algorithm becomes even more valuable. It is $10^{3}$ times better than the ABC algorithm and $10^{2}$ times better than the original BFO algorithm. In addition, since the run length *C* of the BFO and BSO algorithms is reduced in this example, the overall performance of these two algorithms is better than that of the ABC algorithm, further demonstrating that run length is an influential factor affecting the algorithm. The evolution of the average fitness values of the different algorithms is shown in Figure 6. As shown in the figure, the convergence speed of these three algorithms, BFO, BSO, and IBFO, is comparable to and slightly faster than the ABC algorithm.

**Figure 7:**
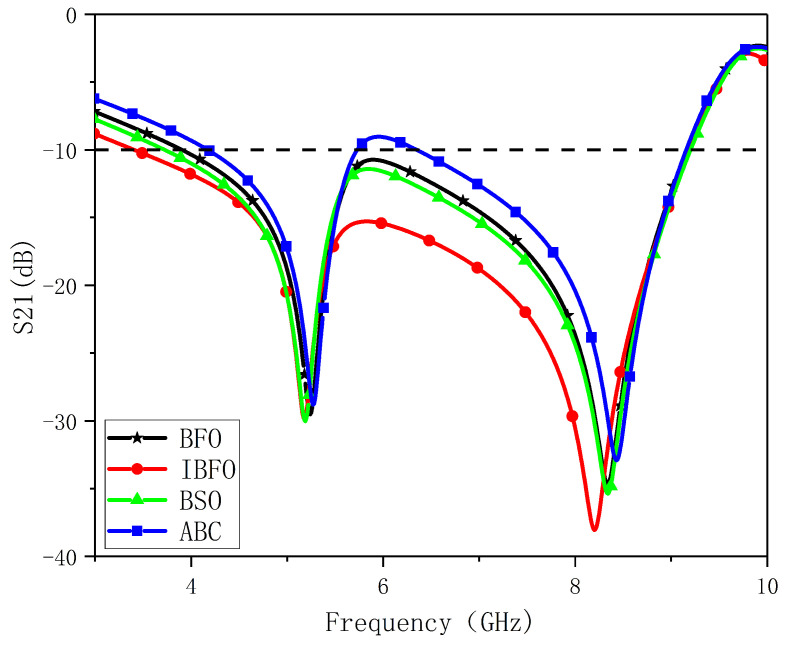
Frequency response of different algorithms.

The optimized structural parameters obtained by running the four algorithms independently 30 times are presented in Table 4. The obtained structural parameters are applied to the FSS shown in Figure 2, and the resulting transmission coefficients are shown in Figure 7. The bandwidth and resonant frequency of the FSS obtained from the IBFO solution satisfy the design objectives. For BFO and BSO algorithms, the FSS bandwidth is smaller than the design target, and the second resonant frequency lagged. Furthermore, the results of the simulation indicate that the structural parameters determined by BFO and BSO are in accordance with the desired bandwidth. Nevertheless, in practice, the transmission coefficient may be greater than −10 dB at the frequency of about 6 GHz due to manufacturing deviations and measurement errors, resulting in the failure to achieve the design goal. The FSS obtained from the ABC solution does not meet the requirement of $S_{21}$ < −10 dB in the target bandwidth, and the second resonant frequency is lagging. In the frequency response of the fractal FSS produced through IBFO optimization, there is a bandwidth between 3.41 and 9.19 GHz, as well as two resonance frequencies of 5.18 and 8.21 GHz. In this paper, the solution error is defined as the difference between the optimized result and the desired value divided by the desired value. The errors of the bandwidth and the first and the second resonance frequencies are 0.345%, 0.385%, and 0.122%, respectively. Within the error range of 0.5%, the design objectives were largely achieved.

## 4. Experimental Verification

**Figure 8:**
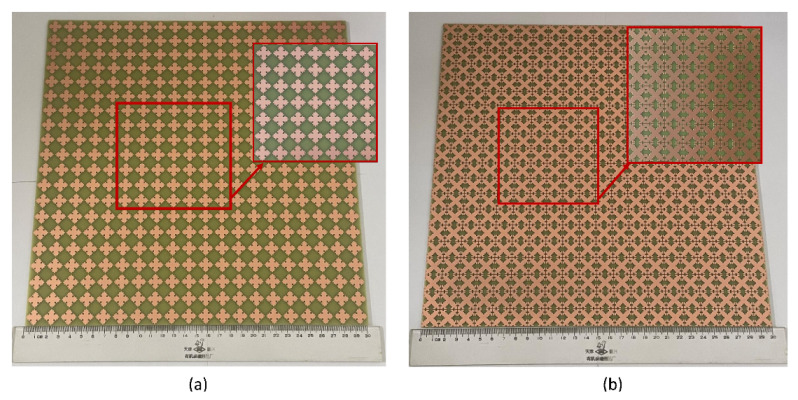
Fabricated optimized fractal FSS. (**a**) Top view, (**b**) Bottom view.

**Figure 9:**
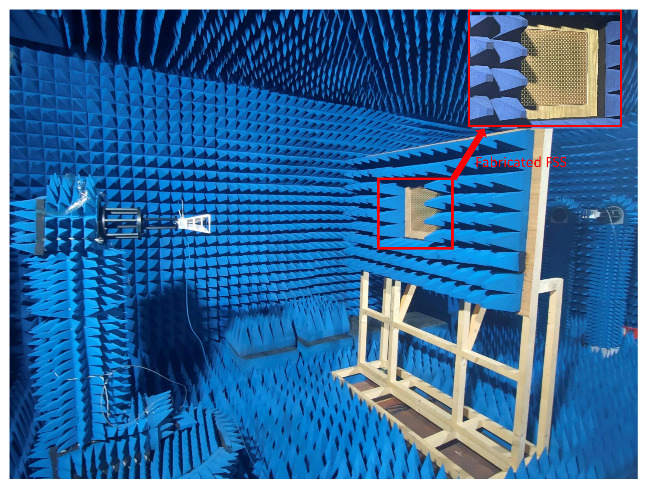
Photograph of the measurement system setup of the fabricated fractal FSS.

For the experimental verification of the simulation results, the fractal FSS optimized by the IBFO algorithm was fabricated. The Koch curve-like metal patch of the top layer is shown in Figure 8a, while the Minkowski island metal patch of the bottom layer is shown Figure 8b. The fractal FSS sample is composed of 20 × 20 FSS units and measures 30 × 30 cm in total. The antenna diffraction’s impact on the test results is diminished since its overall size is greater than the aperture of a typical gain horn. Figure 9 depicts the setup for the FSS test system. The measurement apparatus comprises the proposed fractal FSS samples and two standard gain horns. The FSS is situated between the two horns, which serve as transmitting and receiving antennas. The horns’ polarizations are identical and horizontal.

**Figure 10:**
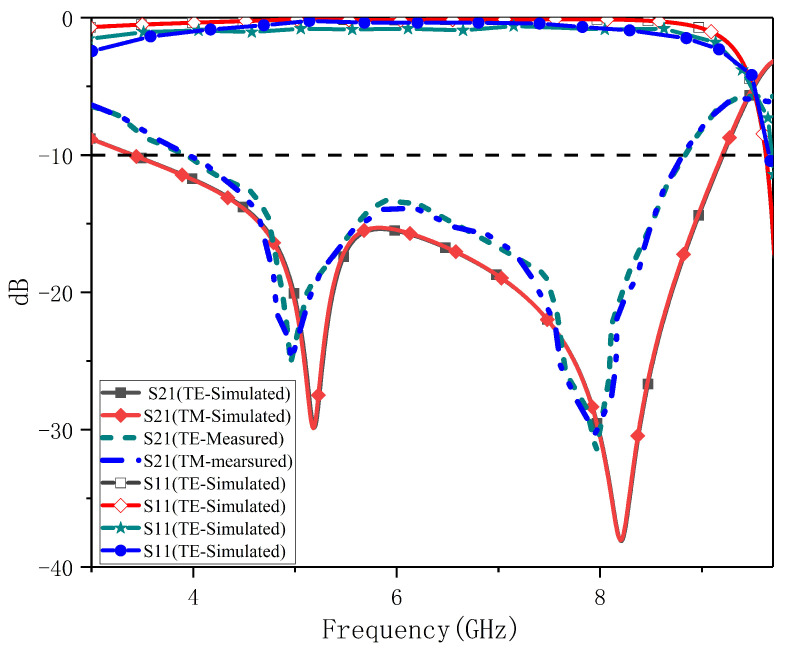
Comparison between the measured and simulated results of the proposed fractal FSS under TE and TM polarization for the normal incidence.

The measurement process consists of two steps: first, the transmission coefficient without the test sample is measured, i.e., the background noise of the measurement environment. Then, the transmission coefficients of the fractal FSS are measured. By normalizing the frequency responses of the two, the frequency response of the fractal FSS is obtained. As shown in Figure 10, the proposed fractal FSS has excellent stability under TE and TM polarization for the normal incident wave (θ=0°) in both simulated and experimental scenarios. Compared with the simulated results, the frequency response obtained from the measurements is essentially identical, except for a small frequency shift due to the manufacturing tolerance and the nonlinear behavior of the substrate.

**Figure 11:**
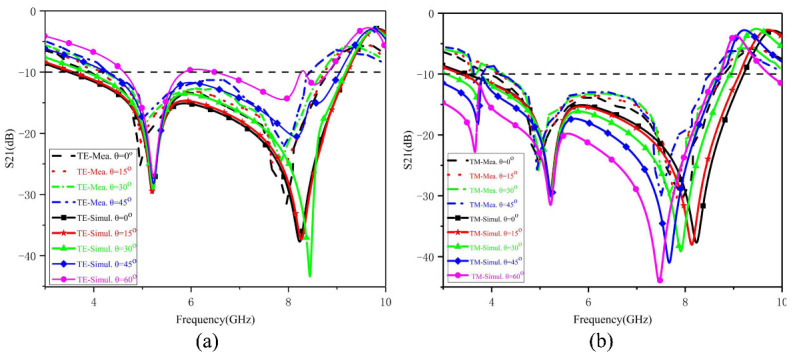
The measured and simulated transmission coefficient of the fractal FSS optimized by IBFO as a function of the oblique incidence angles with (**a**) TE polarization and (**b**) TM polarization.

The transmission coefficients at different incidence angles were simulated and measured to verify the angular stability of the designed FSS. As Figure 11 shows, its frequency filtering characteristics maintain good stability at a 45° oblique incidence angle. The measured frequency response matches the simulation results, verifying the angular stability of the proposed fractal FSS. Under TE polarization, the variation in the incident angle does not have a significant effect on the resonant frequency and bandwidth. The difference between TE and TM polarizations is due to their best-matching impedance in the degree of the oblique incidence angle. Due to the property of angular stability, the proposed fractal FSS is very suitable for radar scattering cross-sections, lenses, sub-reflectors, etc.

Based on the size and broadband performance of the FSS proposed in this paper, it is compared to the FSS proposed in previous studies [39,40,41,42,43,44]. The performance comparison is shown in Table 5. The FSS proposed in ref. [43] has a larger fractional bandwidth than the FSS proposed in this paper, but its oblique incidence angle stability is 15°. Compared with the same type of fractal structure FSS [44], taking into account the fractional bandwidth and oblique incidence angle stability jointly, the proposed FSS achieves better performance. In addition, the proposed FSS has a very thin thickness with 0.025λ and 0.067λ at the lowest and highest frequencies, respectively.

## 5. Conclusions

The design and optimization of fractal FSS using the IBFO algorithm and curve-fitting techniques are proposed in this paper. By varying different structural parameters, the CST software creates databases, and the curve-fitting method determines the relationship between the frequency response of the FSS and the structural parameters. Several enhancements have been made to the BFO algorithm in order to address its inherent flaws, such as chemotaxis, reproduction, elimination, and dispersal. Using the curve-fitting results, the fitness function of the IBFO algorithm can be constructed, resulting in the IBFO algorithm being a powerful tool for synthesizing FSS structures. Blended curve fitting and the IBFO algorithm are proposed to improve the design efficiency without repeating calls to EM simulation software and provide a broad range of application prospects for computer-aided EM design. The fabrication and measurement of the fractal FSS were conducted for the purpose of verifying the effectiveness of the proposed design and optimization method. A broadband fractal FSS with a 3.41–9.19 GHz bandwidth and 5.18 and 8.21 GHz resonant frequencies is achieved. As a fraction of the center frequency at 6.3 GHz, the proposed fractal FSS has a −10 dB fractional bandwidth of 91.7%. Furthermore, the structure has an extremely thin thickness of only 0.025λ and 0.067λ at the lowest and highest frequencies, respectively. Nonetheless, it provides a stable frequency response under the oblique incidence of 45° to both TE and TM polarization. Featuring a low-profile, polarization-insensitive and lightweight, and operating in S, C, and X bands, the proposed structure allows for practical applications in radomes, radars, sub-reflector, lenses, and polarizing grids.

## Figures and Tables

**Table 1:** Initialized parameters for IBFO, BFO, BSO, and ABC.

Parameters	IBFO	BFO	BSO	ABC
Dimensions of search space	2	2	2	2
Total population	26	26	26	26
Chemotactic steps $N_{C}$	50	50	50	-
Limits the length of a swim	4	4	4	-
Reproduction steps $N_{re}$	4	4	4	-
Elimination-dispersal events $N_{ed}$	2	2	2	-
eliminated/dispersed probability $P_{ed}$	0.25	0.25	0.25	-
run length *C*	-	0.005	0.005	-
Weight *w*	0.9	-	0.9	-
Learning rate $\left[c_{1},c_{2}\right]$	[1.2,0.5]	-	[1.2,0.5]	-
depth of the attractant	0.05	0.05	0.05	-
width of the attractant	0.05	0.05	0.05	-
height of the repellent effect	0.05	0.05	0.05	-
width of the repellent effect	0.05	0.05	0.05	-

**Table 2:** Boundary condition of the structural parameters.

Parameters	$d_{1}$	${\mathrm{It}}_{t1}$	${\mathrm{It}}_{t2}$	$b_{1}$	${\mathrm{It}}_{b1}$	${\mathrm{It}}_{b2}$	*p*	*h*
	(mm)			(mm)			(mm)	(mm)
lower limit	3	0.15	0.001	13.5	0.15	0.001	14.5	1.5
upper limit	4.5	0.3	0.125	15	0.35	0.130	16	2.5

**Table 3:** Comparative performance of BFO, BSO, IBFO, and ABC.

Algorithm	Mean Solution	Best Solution	Worst Solution	Standard Deviation
BFO	$7.83\times 10^{-6}$	$1.11\times 10^{-8}$	$1.24\times 10^{-5}$	$5.84\times 10^{-6}$
IBFO	$1.05\times 10^{-7}$	$3.15\times 10^{-10}$	$3.38\times 10^{-6}$	$4.73\times 10^{-7}$
BSO	$3.25\times 10^{-6}$	$8.98\times 10^{-9}$	$1.06\times 10^{-5}$	$6.84\times 10^{-6}$
ABC	$3.45\times 10^{-5}$	$2.01\times 10^{-7}$	$2.45\times 10^{-5}$	$5.30\times 10^{-6}$

**Table 4:** Optimized parameters obtained by different algorithms.

Parameters	$d_{1}$	${\mathrm{It}}_{t1}$	${\mathrm{It}}_{t2}$	$b_{1}$	${\mathrm{It}}_{b1}$	${\mathrm{It}}_{b2}$	*p*	*h*
	(mm)			(mm)			(mm)	(mm)
BFO	3.64	0.302	0.057	14.4	0.259	0.0606	15.2	2.02
IBFO	3.83	0.248	0.081	14.64	0.229	0.0771	15.0	2.20
BSO	3.70	0.27	0.0706	14.47	0.258	0.0610	15.10	2.07
ABC	3.59	0.29	0.0605	14.1	0.256	0.0741	15.2	1.96

**Table 5:** Performance comparison between the proposed fractal FSS and the previously reported broadband FSS.

Ref.	Bandwidth (GHz)	Fractional Bandwidth	Incidence Angle Stability	Thickness (Unit: $\lambda_{0}$)	FSS Structure
[39]	8.76–11.96	30.9%	45°	0.23–0.32	orthogonal dipole resonator
[40]	8.4–18	72.7%	-	0.258–0.492	checkerboard surface
[41]	5.38–12.03	76.4%	20°	0.079–0.176	folded metal strips
[1]	6.25–12	63%	-	0.032–0.061	coding FSS
[42]	7.4–13.4	57.7%	-	0.346–0.493	choked structure
[43]	3.6–11.8	104.91%	15°	0.159–0.524	square loop and rotated cross
[44]	8.8–17.92	68.2%	60°	0.118–0.240	fractal square loop pattern
This paper	3.41–9.18	91.7%	45°	0.025–0.067	Minkowski fractal islands and Koch curve-like

## Data Availability

Not applicable.
